# Case Report: Resolution of radiation pneumonitis with androgens and growth hormone

**DOI:** 10.3389/fonc.2022.948463

**Published:** 2022-08-24

**Authors:** Allen Yen, Kenneth D. Westover

**Affiliations:** Department of Radiation Oncology, University of Texas Southwestern Medical Center, Dallas, TX, United States

**Keywords:** radiation pneumonitis, lung cancer, androgen, growth hormone, steroids anabolics, case report

## Abstract

Radiation pneumonitis (RP) occurs in some patients treated with thoracic radiation therapy. RP often self-resolves, but when severe it is most commonly treated with corticosteroids because of their anti-inflammatory properties. Androgens and human growth hormone (HGH) also have anti-inflammatory and healing properties in the lung, but have not been studied as a remedy for RP. Here we present a case of corticosteroid-refractory RP that resolved with androgen and HGH-based therapy.

**Case Presentation:**

A 62 year old male body builder with excellent performance status presented with locally advanced non-small cell lung cancer characterized by a 7 cm mass in the right lower lobe and associated right hilar and subcarinal lymph node involvement. He was treated with chemoradiation and an excellent tumor response was observed. However, 2 months post-treatment he developed severe shortness of breath and imaging was consistent with RP. His RP was refractory to prednisone and antibiotic therapy, despite various regimens over a 9 month period. The patient self-treated with an androgen and HGH-based regimen and the RP promptly resolved.

**Conclusion:**

The anti-inflammatory properties of androgens and HGH have prompted an exploration of their potential role in therapeutic strategies to treat pro-inflammatory conditions such as sepsis, infections and interstitial lung disease. This case study suggests a potential role for the use of androgens for the treatment of steroid-refractory RP after radiation therapy. However, the applicability of this strategy to general populations should be weighed carefully against secondary effects of these agents, especially in the setting of cancer survivorship.

## Introduction

Chemoradiation is a common treatment for inoperable locally advanced non-small cell lung cancers (1). The treatment involves radiation over 6-7 weeks with concurrent chemotherapy. Side effects can include fatigue, esophagitis, and radiation pneumonitis (RP), with the risk being roughly proportional to the extent of normal tissue exposure (2, 3). In the clinical trials that established chemoradiation as the standard of care, RP was observed in 5-20% of patients and was associated with advanced age or pre-existing lung conditions including chronic obstructive pulmonary lung disease or interstitial lung disease (3–8). At a cellular level, radiation causes several effects including edema, epithelial changes, disruption of the microvasculature, and atelectasis that lead to inflammatory changes resulting in RP (3, 4). Clinically, RP presents weeks to months following treatment, manifesting as fever, cough, shortness of breath, and ground-glass opacities (3, 4). RP can have significant negative impacts on quality of life (4, 9, 10). Severe RP is often treatable with corticosteroids with symptom improvement in 75-93% of patients, but some patients are refractory (3, 11, 12). Androgens are also well known to have anti-inflammatory properties, but have not been studied as a remedy for RP. Older studies have shown that androgen therapy can reduce the risk of exacerbations of asthma and COPD, but given the potential risks of androgen therapy including adverse cardiovascular events and increased risks of malignancy, these medications have not been implemented. In this case report, we present a patient with corticosteroid-refractory RP that responded favorably to androgen-based therapy.

## Case description

**Figure 1:**
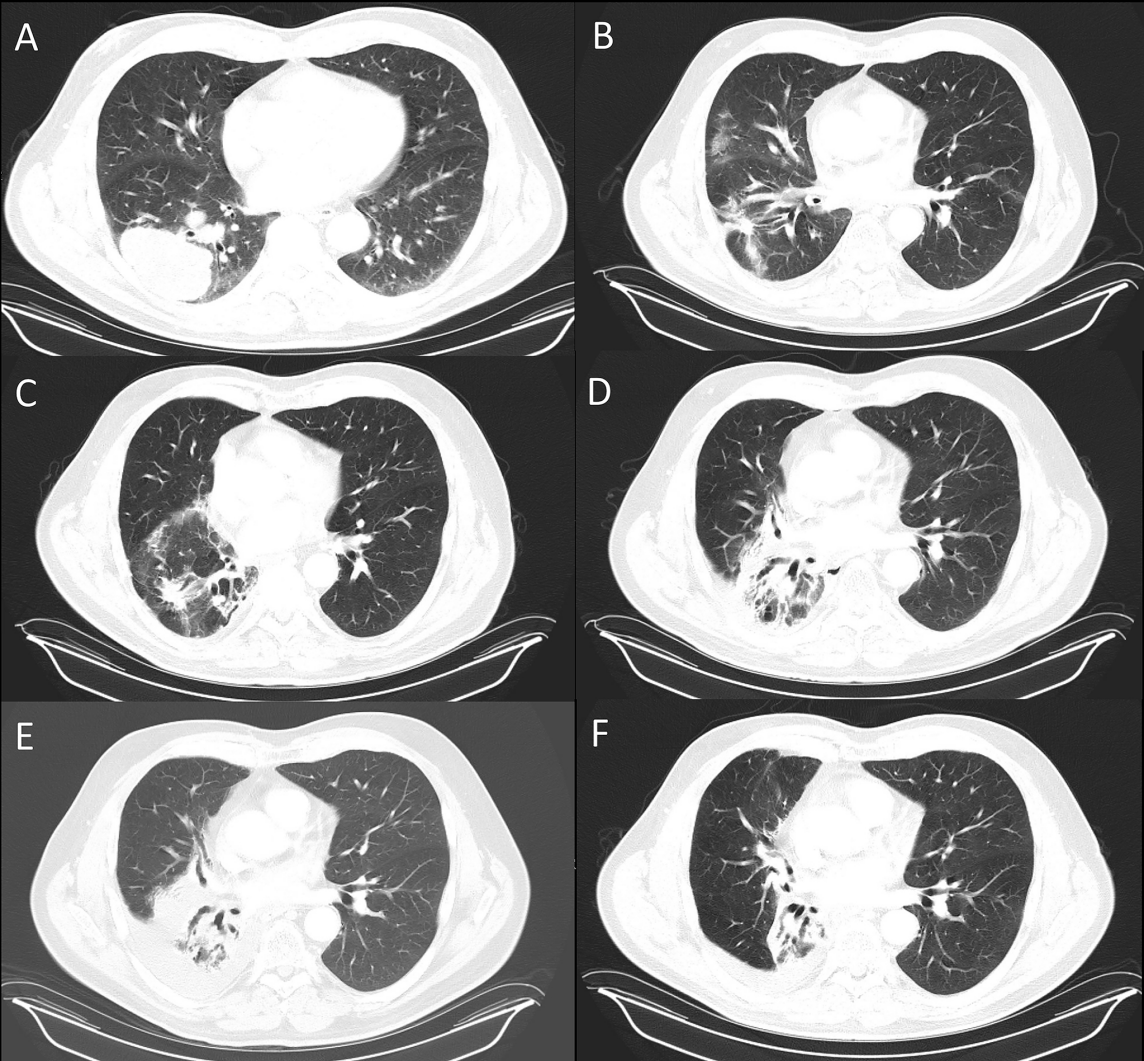
Serial CT’s of the chest shows resolution of lung pneumonitis with anabolic steroids. **(A)** Lung disease at presentation and after completion of definitive chemoRT at **(B)**, 2 months **(C)**, 5 months **(D)**, 8 months **(E)**, 11 months **(F)**, 17 months. The androgen-HGH regimen was initiated after the 12 month scan, **(E)**.

**Figure 2:**
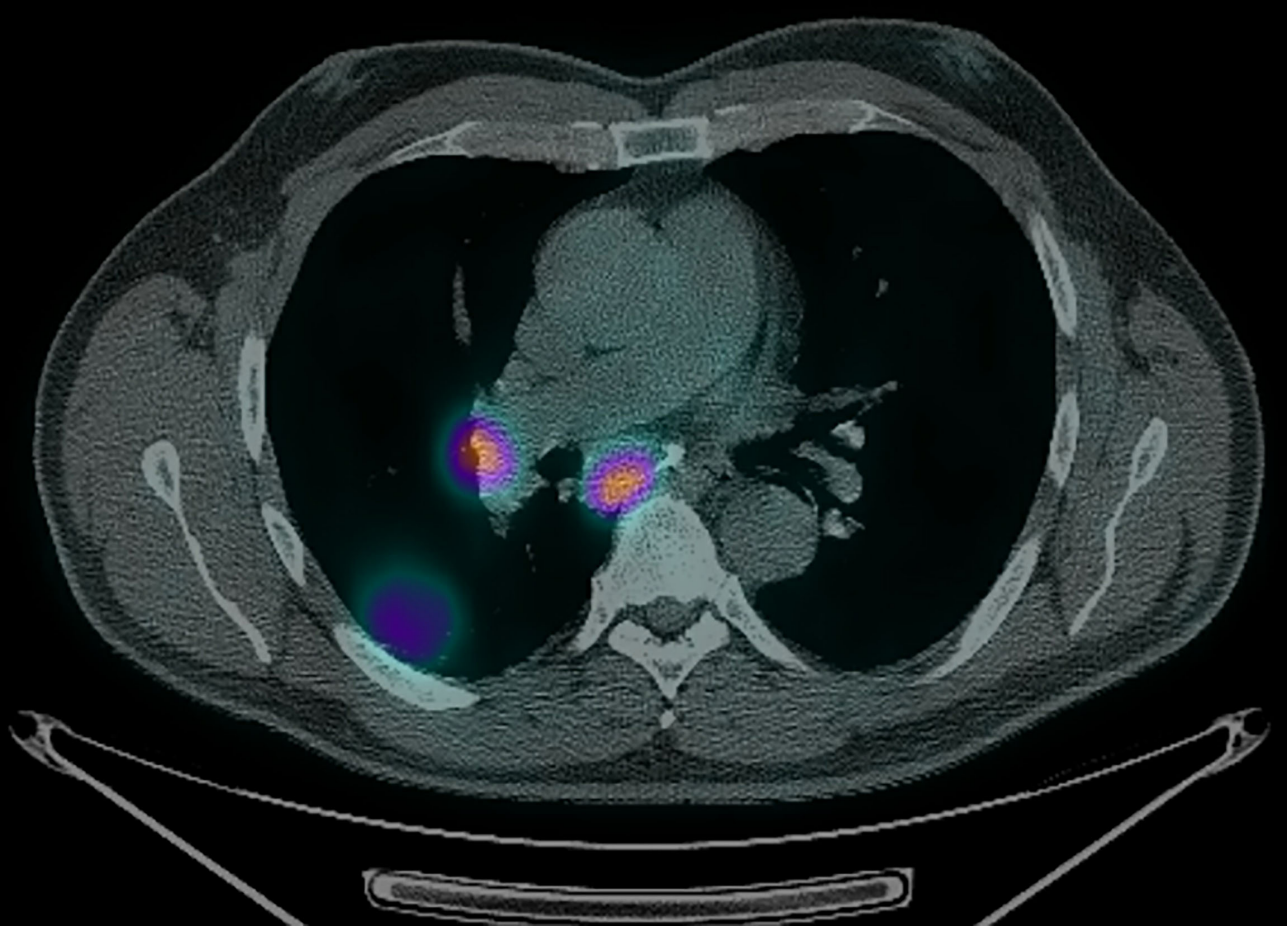
PET/CT showing FDG-avid R hilar and subcarinal lymph nodes.

**Figure 3:**
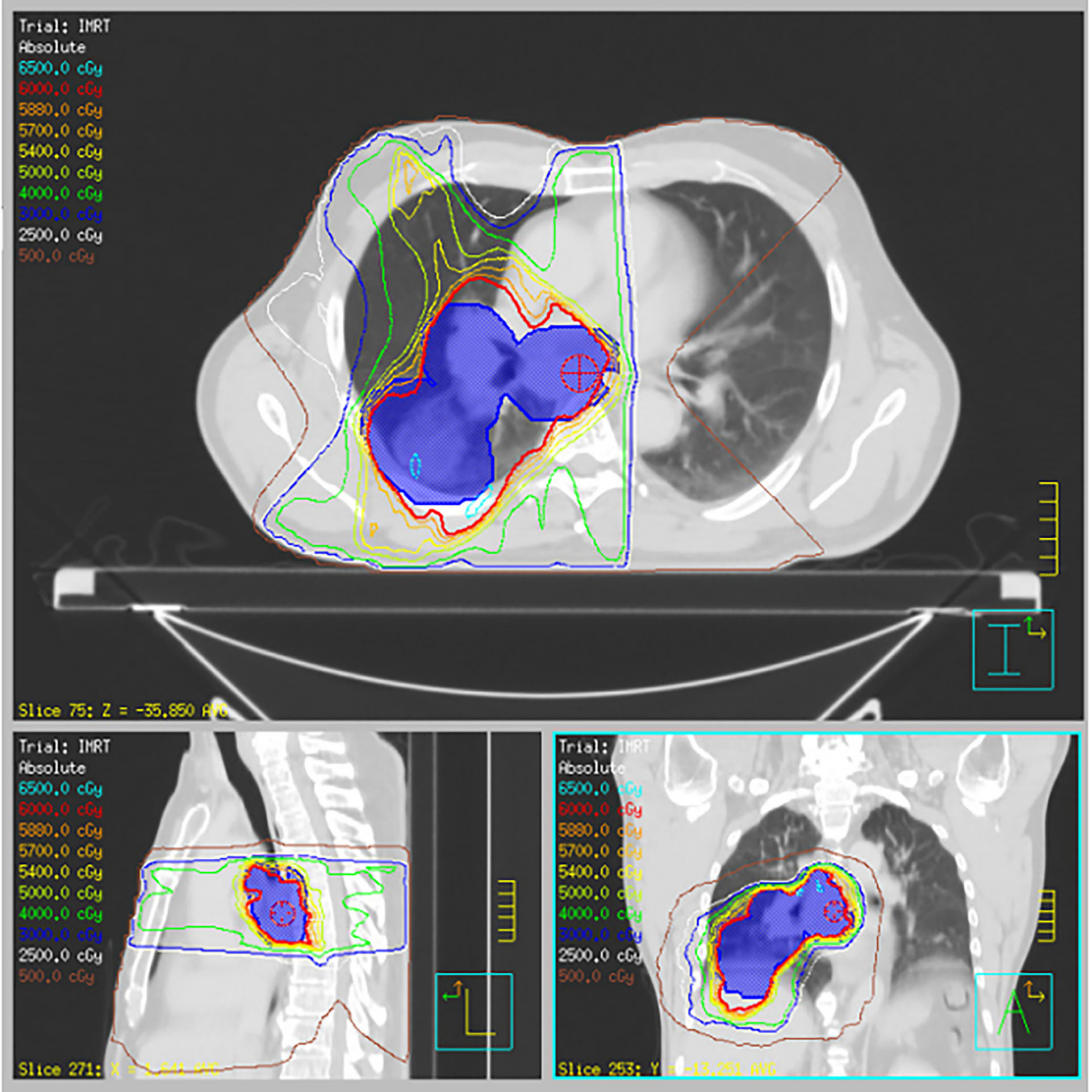
Radiation treatment plan. Representative axial, sagittal, and coronal slices for R lung lesion and mediastinal disease treated to 60 Gy in 30 fractions with concurrent carboplatin and paclitaxel.

A 62-year-old male body builder with a 20-pack year smoking history and surgical history of repaired inguinal hernia and appendectomy presented to his PCP with cough, fatigue, and weight loss. CT of the chest showed a 7 cm mass in the right lower lobe of the lung with enlarged subcarinal lymph nodes (**Figure 1A**). Subsequent PET/CT showed FDG uptake in the lung mass and right hilar and subcarinal lymph nodes (**Figure 2**), but no evidence of metastatic disease. Pre-treatment pulmonary function tests revealed a FVC of 66% and FEV1 of 59%. Biopsy of the right lung mass confirmed poorly-differentiated squamous cell carcinoma of pulmonary origin. Nodal involvement was confirmed by endobronchial ultrasound guided sampling of mediastinal lymph nodes. He was treated with chemoradiation to a dose of 6000 cGy in 30 fractions (**Figure 3**) with 6 cycles of carboplatin and paclitaxel given weekly. Of note, the lung V20 for his radiation plan was 31%. Two cycles of consolidative carboplatin and paclitaxel were also given.

CT Chest 2 months post radiation completion showed shrinkage of the primary mass to 4.4 cm, reduction in the size of involved lymph nodes and associated radiation changes in the surrounding lung (**Figure 1B**). However, by 3 months, he developed malaise and a persistent cough, so an empiric 1 week course of azithromycin and prednisone were prescribed for pneumonia. His symptoms persisted despite therapy and repeat imaging 3 months later showed opacities within the high and intermediate areas of radiation dose consistent with pneumonitis (**Figure 1C**). A 5 week prednisone taper with a starting dose of 25 mg was prescribed. Symptoms improved with therapy but returned as he tapered to 5 mg so the dose was increased to 20 mg and additional course of azithromycin was prescribed. Repeat pulmonary function tests showed had slightly worsened with a FVC of 57% and FEV1 of 63%. Further attempts over the next 4 months to wean from prednisone failed. After 9 months of collective prednisone therapy, he noted abdominal fullness and was found to have elevated ALT and AST of 114 and 53, respectively. On CT imaging he was noted to have hepatic steatosis. Evaluation with colonoscopy, abdominal US, and CT enterography were unremarkable leading to the conclusion that his liver dysfunction was a sequela of steroid therapy. Based on his understanding that androgens can have anti-inflammatory effects, the patient elected to taper prednisone and self-prescribed the androgen-based regimen shown in **Table 1**.

**Table 1:** Anabolic steroid and HGH regimen; CDMT = Chlorodehydromethyltestosterone, QAD = every other day, BID = twice daily, TID = three times daily.

Week 1-8			
Medication Name	Medication Class	Dose	Dosage
Testosterone propionate	AR agonist	250 mg	QAD
Mesterolone	AR agonist	50 mg	BID
Stanozolol	AR agonist	10 mg	TID
Anastrazole	Aromatase inhibitor	0.5 mg	QAD
CDMT	AR agonist	50 mg	BID
Human Growth Hormone	GHR agonist	0.08 mg	Biweekly (Mon and Thurs)
Week 8-16			
**Medication Name**	**Medication Class**	**Dose**	**Dosage**
Testosterone isocaproate	AR agonist	150 mg	Biweekly (Mon and Thurs)
Nandrolone	AR agonist	400 mg	Biweekly (Mon and Thurs)
Boldenone	AR agonist	300 mg	Biweekly (Mon and Thurs)
Anastrazole	Aromatase inhibitor	0.5 mg	QAD
Metandienone	AR agonist	50 mg	BID
Human Growth Hormone	GHR agonist	0.08 mg	Biweekly (Mon and Thurs)
Week 16-20			
**Medication Name**	**Medication Class**	**Dose**	**Dosage**
Testosterone propionate	AR agonist	250 mg	QAD
Drostanolone propionate	AR agonist	100 mg	Biweekly (Mon and Thurs)
Oxandrolone	AR agonist	50 mg	BID
Stanozolol	AR agonist	10 mg	TID
Mesterolone	AR agonist	50 mg	BID
Fluoxymesterone	AR agonist	10 mg	BID
Human Growth Hormone	GHR agonist	0.08 mg	Biweekly (Mon and Thurs)

Within two weeks of initiating therapy, the patient experienced a reduction in abdominal fullness, resolution of cough and improved energy. By eight weeks, he felt fully recovered and was able to return to full workouts lifting weights. During androgen therapy, no other significant changes in medical therapy, diet or lifestyle were noted that could be linked to his improvement. CT of the chest 17 months post radiation therapy showed consolidation of the treated lobe and resolution of pneumonitis elsewhere in the lung (**Figure 1F**). As of most recent follow up, 5 years post treatment, the patient had no clinically significant deficits related to cancer therapy and no evidence of active disease. The patient's treatment course is summarized as as e timeline in the supplementary materials.

## Discussion and conclusions

To our knowledge, this is the first case report of using androgen and HGH-based therapy to treat radiation pneumonitis. However, the concept of treating inflammatory pulmonary disease with androgen or HGH therapy has substantial precedent. The idea that androgens are important regulators of pulmonary inflammation originated with a number of clinical observations. It is well documented that men and women show differences in lung pathology. For example, female neonates produce surfactants earlier in development when compared to male neonates and are at lower risk for respiratory distress syndrome or asthma (13, 14). Also, patients with Klinefelter syndrome, the most common cause of hypogonadism, are 69% more likely to be hospitalized with pulmonary diseases like COPD or pneumonia (15). Additionally, men treated with anti-androgens experience a higher rate of interstitial lung disease with a reported odds ratio up to 6.6 (16). There is also rationale for using HGH to address lung injury. Laboratory studies have shown that HGH may participate in lung development, growth, and repair (17). Additionally, several studies on animal models of acute lung injury have shown dramatic attenuation of lung injury by recombinant HGH (18, 19).

Collectively, these observations have led to prospective studies of androgen therapy and HGH. A study on women with asthma showed that administration of testosterone decreased risks of hospitalizations from asthma attacks by 9.1% in older patients (20).. A meta-analysis from China found that androgens improved body weight, fat-free mass, and symptoms in patients with chronic obstructive pulmonary disease. These changes, however, did not translate into changes in pulmonary function or muscle strength (21). Similarly, another retrospective study of middle-aged men with COPD who received testosterone replacement therapy showed lower rates of respiratory-related hospitalizations (22). A study of HGH in patients with COPD showed improvements in pulmonary function of 27% (23).

The regimen used by the patient in this case report is complex, making it difficult to interpret which agents provided the benefits observed. Nevertheless, the dosages used can be placed into context. The testosterone regimen of 600 mg of testosterone weekly is substantially higher than doses typically used in testosterone replacement therapy (100-200 mg weekly), and has been shown to improve strength and muscle size in healthy men (24). Similarly, doses of nandrolone around 200 mg weekly improved body mass and fat-free mass over the course of 8 weeks (25). This dose was significantly lower than the dose our patient used. The HGH dose of 0.08 mg biweekly is lower than the dose used in the previously cited COPD trial where 0.05 mg/kg was given daily (23). The mesterolone dose of 50 mg BID is similar to the dose used in men with oligospermia and improved sperm counts and mobility who received 100 – 150 mg daily (26). The stanozolol dose of 10 mg TID is lower than the doses used in a recently published trial (2 mg TID) that found improved progression-free survival for patients with high-risk myelodysplastic syndrome (27). In mouse studies, anastrazole was delivered to mice and showed decrease radiation induced lung fibrosis. These mice were given doses equivalent to 1 mg given to an adult daily (28). Although our patients regimen varies from doses reported in literature, there are clear connections between anabolic steroids and beneficial clinical outcomes (29).

In this case, androgen and HGH therapy provided a clear benefit for RP and did not cause cancer recurrence. However, extending this regimen, or a portion of this regimen, to other patients would entail risks, especially within the cancer population. Testosterone or androgen precursors can also increase cardiovascular events, produce neuropsychiatric problems and alter reproductive capacity. Additionally, testosterone has been shown to increase the risk of prostate and testicular cancer and linked to increased risks of breast and liver cancer (30–33). HGH can induce tumor formation in animal models and is associated with increased risk of thyroid and colorectal cancer in patients with acromegaly. However, studies of children and adult cancer survivors who received HGH have not shown an increased risk of cancer progression (34, 35). Clearly, additional prospective studies would be necessary to establish efficacy, dosing and other guidelines for HGH or androgen therapies for RP.

## Data availability statement

The original contributions presented in the study are included in the article/**Supplementary Material**. Further inquiries can be directed to the corresponding author.

## Ethics statement

Written informed consent was obtained from the individual (s) for the publication of any potentially identifiable images or data included in this article.

## Author contributions

AY and KW were responsible for the concept of the case report. AY drafted the manuscript and KW critically revised the manuscript. All authors contributed to the article and approved the submitted version.

## Funding

This work was funded by the Department of Radiation Oncology at UT Southwestern.

## Acknowledgments

We would like to acknowledge the patient presented in this case report for his willingness to assist us with this manuscript.

## Conflict of interest

The authors declare that the research was conducted in the absence of any commercial or financial relationships that could be construed as a potential conflict of interest.

## Publisher’s note

All claims expressed in this article are solely those of the authors and do not necessarily represent those of their affiliated organizations, or those of the publisher, the editors and the reviewers. Any product that may be evaluated in this article, or claim that may be made by its manufacturer, is not guaranteed or endorsed by the publisher.

## References

[1] Ettinger DS Wood DE Aisner DL Akerley W Bauman JR Bharat A Non–small cell lung cancer, version 3.2022, NCCN Clinical practice guidelines in oncology. .Journal of the National Comprehensive Cancer Network (2022) 20(5):497–530. 10.6004/jnccn.2022.0025 35545176

[2] Rodrigues G Lock M D'Souza D Yu E Van Dyk J. Prediction of radiation pneumonitis by dose - volume histogram parameters in lung cancer–a systematic review. Radiother Oncol (2004) 71(2):127–38. doi: 10.1016/j.radonc.2004.02.015 15110445

[3] Bledsoe TJ Nath SK Decker RH. Radiation pneumonitis. Clin Chest Med (2017) 38(2):201–8. doi: 10.1016/j.ccm.2016.12.004 28477633

[4] Jain V Berman AT. Radiation pneumonitis: Old problem, new tricks. Cancers (Basel) (2018) 10(7):222. doi: 10.3390/cancers10070222 PMC6071030 29970850

[5] Vogelius IR Bentzen SM. A literature-based meta-analysis of clinical risk factors for development of radiation induced pneumonitis. Acta Oncol (2012) 51(8):975–83. doi: 10.3109/0284186X.2012.718093 PMC3557496 22950387

[6] Bradley JD Paulus R Komaki R Masters G Blumenschein G Schild S. Standard-dose versus high-dose conformal radiotherapy with concurrent and consolidation carboplatin plus paclitaxel with or without cetuximab for patients with stage IIIA or IIIB non-small-cell lung cancer (RTOG 0617): A randomised, two-by-two factorial phase 3 study. Lancet Oncol (2015) 16(2):187–99. doi: 10.1016/S1470-2045(14)71207-0 PMC4419359 25601342

[7] Curran WJ Jr. Paulus R Langer CJ Komaki R Lee JS Hauser S. Sequential vs. concurrent chemoradiation for stage III non-small cell lung cancer: randomized phase III trial RTOG 9410. J Natl Cancer Inst (2011) 103(19):1452–60. doi: 10.1093/jnci/djr325 PMC3186782 21903745

[8] Socinski MA Zhang C Herndon JE 2nd Dillman RO Clamon G Vokes E. Combined modality trials of the cancer and leukemia group b in stage III non-small-cell lung cancer: Analysis of factors influencing survival and toxicity. Ann Oncol (2004) 15(7):1033–41. doi: 10.1093/annonc/mdh282 15205196

[9] Trott KR Herrmann T Kasper M. Target cells in radiation pneumopathy. Int J Radiat Oncol Biol Phys (2004) 58(2):463–9. doi: 10.1016/j.ijrobp.2003.09.045 14751516

[10] Kanemoto A Matsumoto Y Sugita T. Timing and characteristics of radiation pneumonitis after stereotactic body radiotherapy for peripherally located stage I lung cancer. Int J Clin Oncol (2015) 20(4):680–5. doi: 10.1007/s10147-014-0766-3 25373854

[11] Henkenberens C Janssen S Lavae-Mokhtari M Leni K Meyer A Christiansen H. Inhalative steroids as an individual treatment in symptomatic lung cancer patients with radiation pneumonitis grade II after radiotherapy – a single-centre experience. Radiat Oncol (2016) 11(1):12. doi: 10.1186/s13014-016-0580-3 26830686 PMC4736495

[12] Sekine I Sumi M Ito Y Nokihara H Yamamoto N Kunitoh H. Retrospective analysis of steroid therapy for radiation-induced lung injury in lung cancer patients. Radiother Oncol (2006) 80(1):93–7. doi: 10.1016/j.radonc.2006.06.007 16820236

[13] Farrell PM Wood RE. Epidemiology of hyaline membrane disease in the united states: Analysis of national mortality statistics. Pediatrics (1976) 58(2):167–76. 951131

[14] Myers T. Pediatric asthma epidemiology: incidence, morbidity, and mortality. Respir Care Clinics North America (2000) 6:1–14. doi: 10.1016/S1078-5337(05)70054-X 10639553

[15] Bojesen A Juul S Birkebaek NH Gravholt CH. Morbidity in klinefelter syndrome: a Danish register study based on hospital discharge diagnoses. J Clin Endocrinol Metab (2006) 91(4):1254–60. doi: 10.1210/jc.2005-0697 16394093

[16] Nawa H Niimura T Hamano H Yagi K Goda M Zamami Y. Evaluation of potential complications of interstitial lung disease associated with antiandrogens using data from databases reporting spontaneous adverse effects. Front Pharmacol (2021) 12:655605. doi: 10.3389/fphar.2021.655605 34177574 PMC8220081

[17] Zhang C Cai R Lazerson A Delcroix G Wangpaichitr M Mirsaeidi M. Growth hormone-releasing hormone receptor antagonist modulates lung inflammation and fibrosis due to bleomycin. Lung (2019) 197(5):541–9. doi: 10.1007/s00408-019-00257-w PMC6778540 31392398

[18] Yi C Cao Y Mao SH Liu H Ji LL Xu SY. Recombinant human growth hormone improves survival and protects against acute lung injury in murine staphylococcus aureus sepsis. Inflamma Res (2009) 58(12):855–62. doi: 10.1007/s00011-009-0056-0 19536455

[19] Yi C Wang SR Zhang SY Yu SJ Jiang CX Zhi MH. Effects of recombinant human growth hormone on acute lung injury in endotoxemic rats. Inflammation Res (2006) 55(11):491–7. doi: 10.1007/s00011-006-6011-4 17122967

[20] Wulfsohn NL Politzer WM Henrico JS. TESTOSTERONE THERAPY IN BRONCHIAL ASTHMA. S Afr Med J (1964) 38:170–2. 14127213

[21] Pan L Wang M Xie X Du C Guo Y. Effects of anabolic steroids on chronic obstructive pulmonary disease: a meta-analysis of randomised controlled trials. PloS One (2014) 9(1):e84855. doi: 10.1371/journal.pone.0084855 24427297 PMC3888411

[22] Baillargeon J Urban RJ Zhang W Zaiden MF Javed Z Sheffield-Moore M. Testosterone replacement therapy and hospitalization rates in men with COPD. Chron Respir Dis (2019) 16:1479972318793004. doi: 10.1177/1479972318793004 30205698 PMC6302963

[23] Pape GS Friedman M Underwood LE Clemmons DR. The effect of growth hormone on weight gain and pulmonary function in patients with chronic obstructive lung disease. Chest (1991) 99(6):1495–500. doi: 10.1378/chest.99.6.1495 2036835

[24] van Amsterdam J Opperhuizen A Hartgens F. Adverse health effects of anabolic–androgenic steroids. Regul Toxicol Pharmacol (2010) 57(1):117–23. doi: 10.1016/j.yrtph.2010.02.001 20153798

[25] van Marken Lichtenbelt WD Hartgens F Vollaard NB Ebbing S Kuipers H. Bodybuilders' body composition: effect of nandrolone decanoate. Med Sci Sports Exerc (2004) 36(3):484–9. doi: 10.1249/01.MSS.0000117157.06455.B0 15076791

[26] Varma TR Patel RH. The effect of mesterolone on sperm count, on serum follicle stimulating hormone, luteinizing hormone, plasma testosterone and outcome in idiopathic oligospermic men. Int J Gynaecol Obstet (1988) 26(1):121–8. doi: 10.1016/0020-7292(88)90206-8 2892728

[27] Liu Y Yang C Xue H Ye F Sun W Wang J. Stanozolol improves the progression-free survival of patients with high-risk myelodysplastic syndrome after decitabine treatment. Int J Hematol (2021) 113(6):807–14. doi: 10.1007/s12185-021-03115-9 33646527

[28] Bese NS Altinok AY Ozsahin EM Yildirim S Sut N Altug T. Aromatase inhibitors and radiation-induced lung fibrosis. J Clin Oncol (2008) 26(15_suppl):614. doi: 10.1200/jco.2008.26.15_suppl.614 27326979

[29] McCullough D Webb R Enright KJ Lane KE McVeigh J Stewart CE. How the love of muscle can break a heart: Impact of anabolic androgenic steroids on skeletal muscle hypertrophy, metabolic and cardiovascular health. Rev Endocr Metab Disord (2021) 22(2):389–405. doi: 10.1007/s11154-020-09616-y 33269425 PMC8087567

[30] Synder P. Use of androgens and other hormones by athletes (2020) (Accessed November 19, 2021).

[31] Bianchi VE. The anti-inflammatory effects of testosterone. J Endocr Soc (2018) 3(1):91–107. doi: 10.1210/js.2018-00186 30582096 PMC6299269

[32] Mohamad NV Wong SK Wan Hasan WN Jolly JJ Nur-Farhana MF Ima-Nirwana S. The relationship between circulating testosterone and inflammatory cytokines in men. Aging Male (2019) 22(2):129–40. doi: 10.1080/13685538.2018.1482487 29925283

[33] Salerno M Cascio O Bertozzi G Sessa F Messina A Monda V. Anabolic androgenic steroids and carcinogenicity focusing on leydig cell: a literature review. Oncotarget (2018) 9(27):19415–26. doi: 10.18632/oncotarget.24767 PMC5922407 29721213

[34] Clayton PE Banerjee I Murray PG Renehan AG. Growth hormone, the insulin-like growth factor axis, insulin and cancer risk. Nat Rev Endocrinol (2011) 7(1):11–24. doi: 10.1038/nrendo.2010.171 20956999

[35] Jenkins PJ Mukherjee A Shalet SM. Does growth hormone cause cancer? Clin Endocrinol (Oxf) (2006) 64(2):115–21. doi: 10.1111/j.1365-2265.2005.02404.x 16430706

