# Supplementary figures and images for: Case Report: Resolution of radiation pneumonitis with androgens and growth hormone
Source: Front Oncol. 2022 Aug 24;12:948463. doi: 10.3389/fonc.2022.948463 (PMC9449808; doi:10.3389/fonc.2022.948463)

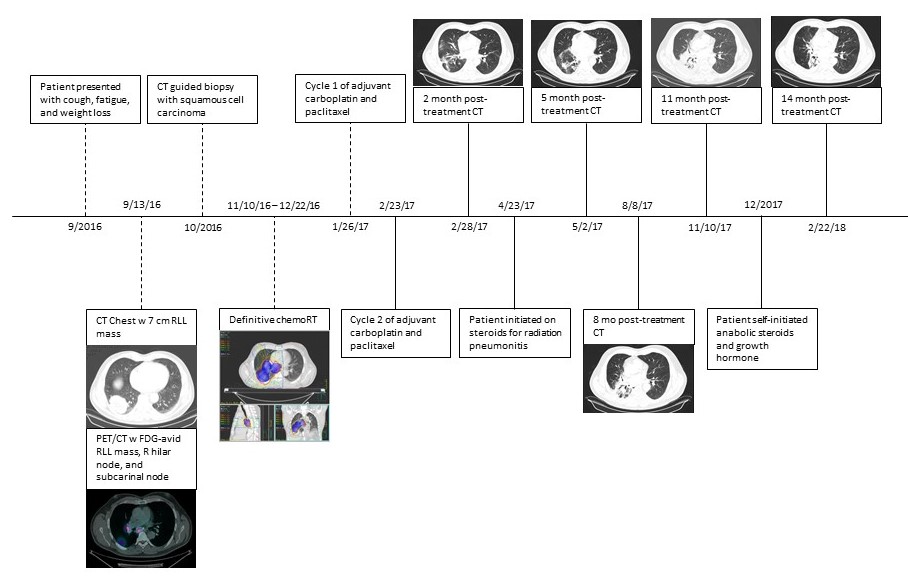

Supplement: Supplementary file 1 [file Image_1.jpg]
